# The Neuroimmune Connectome: Understanding how the Peripheral Nervous System shapes Immunity

**DOI:** 10.1093/immhor/vlaf080

**Published:** 2026-04-22

**Authors:** Shanel M Tsuda, Anna-Maria Globig

**Affiliations:** Allen Institute for Immunology, Seattle, WA, United States; Allen Institute for Immunology, Seattle, WA, United States

**Keywords:** cancer, infection, neuroimmunology

## Abstract

Nerves are active regulators of immunity. Here, we review the current understanding of neuroimmune communication, focusing on how peripheral neurons instruct immune cell differentiation and function. We discuss mechanisms by which neurotransmitters, neuropeptides, and cytokines mediate reciprocal signaling between nerves and immune cells across infection, allergy, autoimmunity, and cancer. We introduce the concept of nerves as “state-setters” of immunity and summarize emerging therapeutic strategies that target neuroimmune pathways. Finally, we highlight current methodological challenges and outline priorities for mapping the peripheral neuroimmune connectome to accelerate clinical translation.

**Figure:**
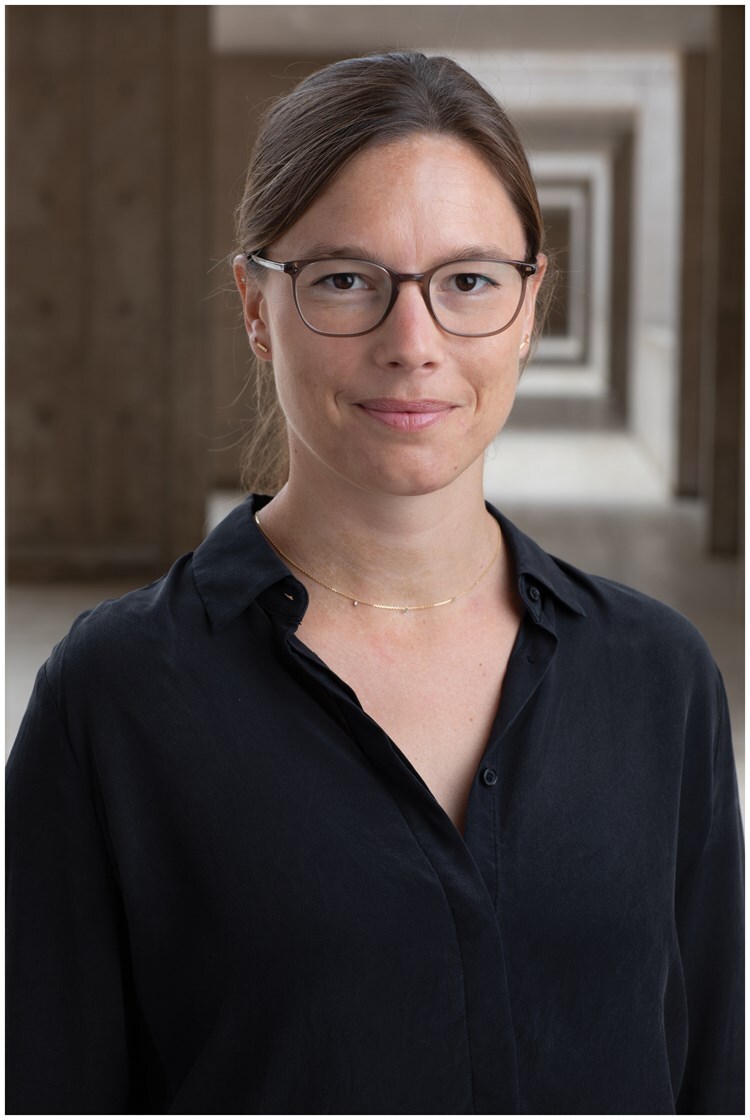
Anna-Maria Globig is an Assistant Investigator at the Allen Institute, where she studies how neuroimmune interactions shape immune responses in viral infection, cancer, and autoimmunity. Anna earned her medical degree from the Albert-Ludwigs-University in Freiburg, conducting her doctoral research in Robert Thimme's lab on the immunopathogenesis of inflammatory bowel disease. After clinical training in Internal Medicine, she joined Susan Kaech's lab at the Salk Institute as a Postdoctoral Fellow. In her postdoctoral work, she discovered a sympathetic nerve-to-T cell signaling pathway that suppresses antiviral and antitumor immunity, revealing a therapeutic opportunity to enhance the efficacy of existing cancer immunotherapies.

## Introduction

The nervous and immune systems are the body’s two main surveillance networks. However, despite their many similarities, including the ability to sense and react to internal and environmental stimuli, they have historically been studied in isolation. It is only in recent years that we are starting to uncover the rules and mechanisms of their bidirectional crosstalk, and the many molecular mediators and receptors involved in this conversation. This is particularly true for peripheral neuroimmunology, a rapidly emerging field that is only beginning to take shape.

**Figure 1.**
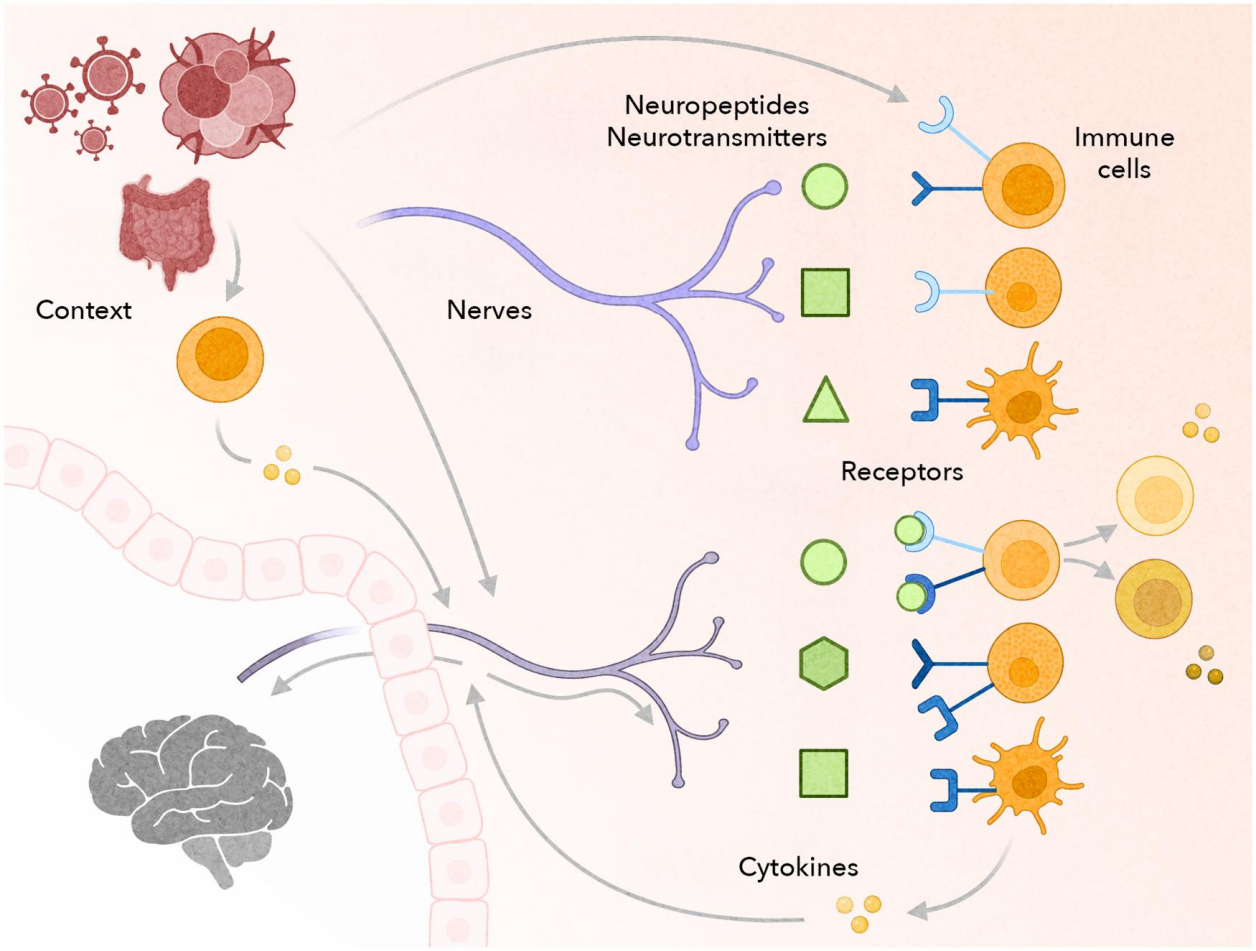
Neuroimmune communication is complex. Peripheral tissues are innervated by multiple types of nerves, each capable of releasing multiple different neurotransmitters and neuropeptides. Immune and nonimmune cells express a broad repertoire of neurotransmitter and neuropeptide receptors whose expression varies with tissue context, immune challenge, and cellular differentiation state. The resulting signaling pathways are bidirectional and functionally nonredundant, enabling neuronal inputs to selectively shape immune cell activation, differentiation, and function.

Neuroimmune communication is inherently complex. Both systems comprise diverse cell types, multiple types of nerves innervate normal and abnormal tissues, and each neurotransmitter can act through several receptors. These receptors are differentially expressed depending on immune cell state, and may be functionally nonredundant. Moreover, neurotransmitter and neuropeptide receptors are expressed by many different immune and nonimmune cells, making it challenging to discern cell type intrinsic effects. Conversely, immune-derived signals like cytokines can shape neuronal function, influencing sensations like pain or itch (Fig. 1).

Crucially, neuroimmune interactions often occur in defined anatomical “hotspots”, in which nerves and immune cells are positioned in close proximity to each other, both within normal tissues like skin, gut, and lymphoid organs and in abnormal tissues like tumors.^1–5^ At these sites, neural inputs regulate key immunological processes, from innate and adaptive responses to infection and cancer, to dysregulated immune activity in autoimmunity. Understanding the mechanisms of neuroimmune crosstalk is not merely of academic interest; it carries immediate translational promise. Targeting neuroimmune pathways could yield new treatment strategies for immune-related diseases, in some cases by repurposing drugs already approved for other indications. While this review focuses on peripheral neuroimmunology, peripheral immune activity is critically shaped by continuous bidirectional communication with the central nervous system (CNS) through central-peripheral feedback loops.^6–8^

In this article, we synthesize recent insights into neuroimmune interactions across tissue and disease contexts, introduce the concept of nerves as “state setters” of immunity, and explore the potential of neuroimmune targeted drugs as precision immunotherapy. Finally, we highlight key challenges and opportunities on the horizon for the rapidly growing field of peripheral neuroimmunology.

## Neuroimmune interactions during immune challenges

While prior work has shed light on how neurotropic infections impact the CNS, much less is known about neuroimmune interactions in peripheral tissues, especially in the context of immune challenges.^9–12^ Increasing evidence suggests that peripheral nerves are not merely passive bystanders but active regulators of local immunity, shaping inflammatory tone and leukocyte recruitment during infection. Understanding these pathways is critical, as the same neuronal signals that coordinate protective immune responses may also amplify pathology when dysregulated. The mechanisms of this neuroimmune crosstalk can be illustrated through well-characterized infection models, each revealing distinct strategies by which nerves and immune cells communicate to balance host defense and tissue homeostasis. The following sections highlight these interactions across cancer, viral, helminth, and bacterial infections, as well as in allergic and autoimmune contexts, underscoring the context-dependent nature of peripheral neuroimmunology.

### Neuroimmune interactions in cancer

Tumor innervation and neuronal activity within tumors are increasingly recognized as critical regulators of the tumor microenvironment (TME). Indeed, emerging evidence suggests that sustained exposure to a cocktail of different neural-derived factors may progressively shape the TME in ways that permit tumors to evade immunosurveillance.^9^^,^^13^ In addition to signaling via neurotransmitters and neuropeptides, nerves can also express immune checkpoint molecules such as PD-1 and PD-L1.^14^

Although nerves in tumors were first described more than a century ago,^15–18^ we are only starting to appreciate more recently that hijacking nerves is another mechanism cancers use to promote their growth and metastasis, analogous to the well-established concept of tumors hijacking vasculature.^19^

Certain tumor types, including colorectal cancer, prostate cancer, and pancreatic ductal adenocarcinoma, are particularly highly innervated, with innervation driving tumor initiation, proliferation, invasion, and metastasis, and correlating with poorer clinical outcomes.^13^^,^^20–28^ Many tumors also produce neurotrophins like nerve growth factor (NGF), which not only increase tumor innervation, but also directly boost the production of neuropeptides.^29^^,^^30^

Beyond direct neuronal influence on cancer cells, the crosstalk between the nervous and immune systems within the TME has gained increasing attention in recent years,^9^^,^^13^^,^^22^^,^^31^ as it has become clear that tumor-infiltrating immune cells express receptors for neurotransmitters and neuropeptides, enabling them to detect neuronal signals.^2^^,^^32–34^ Studies of the TME have mainly centered on sympathetic and sensory nerves:

Sympathetic nerves regulate antitumor immunity at multiple levels. They restrict T cell infiltration by inhibiting egress from lymph nodes,^35^ modulate T cell activation,^36^ and drive CD8^+^ T cells toward terminal exhaustion through noradrenaline signaling via the beta 1 adrenergic receptor ADRB1 on tumor-infiltrating lymphocytes. ADRB1 deletion or pharmacological blockade of this pathway prevents exhaustion and restores T cell effector function.^2^ Sympathetic nerves also regulate the immunosuppressive myeloid compartment, as noradrenaline receptor deletion in myeloid-derived suppressor cells reduces tumor growth, PD-L1 expression, and systemic levels of immunosuppressive cytokines.^34^ These findings position sympathetic nerves as central regulators of both T cell dysfunction and the broader immunosuppressive TME.

In parallel, sensory neurons have emerged as key regulators of cancer progression. The sensory nervous system directly impacts cancer immunity, primarily through nociceptor neurons that release key neuropeptides upon activation. In melanoma, nociceptors and calcitonin gene-related peptide (CGRP) affect immunosurveillance by promoting T cell exhaustion through RAMP1 expression on CD8^+^ T cells.^37^ In addition to promoting immunosuppression, sensory nerve derived neuropeptides such as CGRP and substance P (SP) have been shown to increase tumor cell survival and metastasis directly.^28^^,^^30^

Finally, immune cells themselves can secrete neurotransmitters, contributing to non-neuronal neurotransmitter signaling that shapes the TME and impacts cancer progression. Notable examples of this include T cell–derived acetylcholine (ACh), which can regulate other immune cell types like macrophages, dendritic cells (DCs), B cells and natural killer cells^38^^,^^39^; B cell–derived Gamma-Aminobutyric Acid, which promotes anti-inflammatory macrophage phenotypes and T cell suppression^40^; and platelet-derived serotonin, which reduces T cell effector function.^41^

Together, these findings establish tumor innervation and neuroimmune crosstalk as central components of the TME, in which reciprocal communication between nerves, immune cells, and cancer cells shapes antitumor immune responses and clinical outcomes.

### Viral infections

#### Influenza A

Peripheral infections can influence CNS function through neuroimmune pathways, even in the absence of direct viral neurotropism, and influenza A virus (IAV) provides an example of this phenomenon. While certain strains, such as H1N1, are known to cause neuropathies in humans, most IAV strains are non-neurotropic, suggesting that associated neuroinflammation likely arises secondary to peripheral infection. Indeed, peripheral IAV infection in mice induces hippocampal neuroinflammation, leading to cognitive deficits and elevated levels of proinflammatory cytokines.^42^

In addition to peripheral infections influencing the CNS, reciprocal neuroimmune communication in peripheral tissues—such as sympathetic nervous system signaling—modulates antiviral immunity. Stress can profoundly impact antiviral immunity, with effects that depend on its type, timing, and duration.^43^ In mouse models, daily restraint stress during IAV infection reduces T and B cell recruitment to draining lymph nodes, resulting in higher viral titers.^44^ Sympathetic nerve activity further suppresses antiviral defense by impairing the capacity of antigen-presenting cells to activate naïve CD8^+^ T cells, and limiting the expansion of CD4^+^ and CD8^+^ T cells through the beta 2 adrenergic receptor ADRB2.^45–47^ In contrast, a single exposure to stress before vaccination was shown to enhance T and B cell responses.^48^ Thus, while acute stress can transiently boost immunity, chronic stress appears broadly immunosuppressive, and the balance between these opposing effects warrants further investigation.^49^^,^^50^ Of note, stress modulates immune function through 2 principal pathways: (i) the hypothalamic-pituitary-adrenal axis triggers glucocorticoid release, which potently suppresses immune activity and is widely leveraged clinically as an immunosuppressive strategy^51^; and (ii) the sympathetic nervous system drives the release of the stress hormones adrenaline and noradrenaline.^7^^,^^52^ The individual contributions of these two axes can be difficult to experimentally disentangle.

Additionally, sensory nerves also play an important role in shaping peripheral immune responses against IAV infection. Ablation of Transient Receptor Potential Vanilloid 1 (TRPV1)^+^ and Nav1.8^+^ nociceptors reduces survival of IAV-infected mice and increases viral spread and lung pathology.^53^ Loss of TRPV1^+^ nociceptors impairs interferon signaling in myeloid cells and increases expansion of neutrophils and monocyte-derived macrophages, highlighting the role of nociceptors as regulators of viral spread and pathogenic myeloid cell states during IAV infection.

Recent studies have further uncovered a mechanism of cellular communication in which B cells act as a source of the neurotransmitter ACh. Following IAV infection, choline acetyltransferase (ChAT)^+^ B cells produce ACh, which acts on α7nAChR^+^ macrophages to suppress TNFα secretion and limit inflammation. Selective deletion of ChAT in B cells disrupts this pathway, resulting in heightened inflammation and increased infiltration of CD8^+^ T cells.^54^ Together, these findings illustrate that neuronal signaling mechanisms profoundly shape antiviral immunity during influenza infection.

#### Herpes simplex virus

Neurotropic viral infections exemplify how direct infection of the nervous system alters neuroimmune homeostasis and shapes host immune responses. Herpes simplex virus 1 (HSV-1), HSV-2, and human immunodeficiency virus (HIV) can infect sensory neurons and establish persistent infection within the dorsal root ganglia (DRG).^55^ In HSV infection, this clinically results in localized disease corresponding to the innervated dermatome, manifesting as painful lesions in patients.^56^^,^^57^ During the latent phase of HSV infection, antigen-specific CD8^+^ T cells in the DRG suppress viral reactivation by producing IFNγ.^58^ However, stress can impair this CD8^+^ T cell surveillance of latently infected neurons, thereby triggering viral reactivation.^59^ Emerging evidence additionally indicates that sensory nerves themselves are active modulators of the immune response to HSV-1. Loss of Nav1.8^+^ sensory nerves increases neutrophil recruitment and lesion size following HSV infection, while impairing the CD8^+^ T cell response, a phenotype rescued by neutrophil depletion.^60^ Together, these findings highlight that neuronal circuits regulate the immune response to neurotropic viral infection, impacting viral control and tissue pathology.

#### Lymphocytic choriomeningitis virus

Lymphocytic choriomeningitis virus (LCMV) is an established model for studying antiviral T cell responses and T cell-mediated neuroinflammation. Intracerebral LCMV infection in mice induces fatal choriomeningitis, driven almost exclusively by infiltrating CD8^+^ T cells recruited from the periphery,^61^ demonstrating that CNS-derived antigens can effectively prime peripheral adaptive immune responses. Conversely, peripheral LCMV infection has been shown to generate antigen-specific tissue-resident memory T cells in the brain.^62^^,^^63^ Consistent with this, virus-specific T cells recognizing IAV, Epstein-Barr virus, and cytomegalovirus have been detected in the brains of glioblastoma patients, suggesting that prior systemic infections can drive T cell trafficking to the CNS.^64^ Despite these observations, the pathways through which peripheral infections influence CNS immunity, and how this is impacted by the crosstalk between nerves and immune cells remain poorly understood.

In acute LCMV infection, neuronal signaling via CGRP has been shown to influence immune cell differentiation and function: Receptor Activity-Modifying Protein 3 (RAMP3), a subunit of the CGRP receptor, differentially regulates T helper cell polarization,^65^ inhibiting T helper 2 (Th2) cell differentiation, while promoting Th1 cell development, enhancing IFNγ production, and augmenting CD8^+^ T cell mediated antiviral immunity. During chronic LCMV infection, sympathetic nervous system activity similarly exerts a critical influence on T cell fate. Exhausted CD8^+^ T cells upregulate the noradrenaline receptor ADRB1 and localize near sympathetic nerves in an ADRB1-dependent manner.^2^ Noradrenaline signaling through ADRB1 suppresses T cell cytokine production and proliferation, whereas loss of ADRB1 prevents terminal T cell exhaustion.

In addition, cholinergic signaling also contributes to T cell migration and functional regulation. Following LCMV infection, activated virus-specific CD4^+^ and CD8^+^ T cells express ChAT, enabling ACh production that promotes vasodilatation and T cell trafficking into infected tissues.^66–68^ During chronic infection, ChAT^+^ CD8^+^ T cells highly express the inhibitory receptors PD-1, TIM-3, and LAG3.^68^ How ACh signaling impacts distinct states of T cell exhaustion during chronic infection remains to be elucidated. Importantly, direct comparative analyses of neuronal signaling axes across acute and chronic LCMV infection are needed to delineate how distinct neuroimmune cues drive divergent T cell fates in different infection contexts, and how immune signals, in turn, modulate neuronal activity.

### Bacterial infections

#### Staphylococcus aureus

*Staphylococcus aureus* infection engages sensory neurons as active participants in host defense. Stimulation of TRPV1^+^ sensory nerves is sufficient to elicit a type 17 immune response in the skin through release of CGRP, and activation of TRPV1^+^ neurons prior to infection enhances host protection against *S. aureus* and *Candida albicans.*^69^ Additionally, it has been shown that *S. aureus* activates Nav1.8^+^ sensory neurons during skin infections. Depletion of these neurons prior to infection reduced pain responses but led to exaggerated footpad swelling and increased infiltration of innate and adaptive immune cells into the draining lymph node.^70^ Granulocytes were found to colocalize with Nav1.8^+^ neurons, and both neutrophils and macrophages formed close contacts with DRG neurons, implicating them as key mediators of this neuroinflammatory response. In contrast, TRPV1^+^ neurons have also been shown to suppress host defense against lethal *S. aureus*–induced pneumonia by suppressing cytokine production through CGRP. Nociceptor deletion led to improved outcomes of infection, but this effect was reversed in γδ T cell–deficient mice, suggesting a mechanism of neuronal suppression dependent on γδ T cells.^71^

Together, these findings demonstrate that bacterial infection can activate sensory nerve circuits, which in turn modulate both innate and adaptive immune responses, ultimately balancing antimicrobial defense and inflammatory pathology.

#### Salmonella typhimurium

During *Salmonella* infection, neuroimmune communication in the gut plays a crucial role in coordinating tissue protection and antimicrobial defense. Activation of extrinsic sympathetic neurons leads to noradrenaline release, which acts on ADRB2^+^ muscularis macrophages, driving tissue protective macrophage phenotypes and safeguarding intestinal homeostasis.^72^ Notably, these macrophages are also essential for protection of enteric neurons during both bacterial and helminth infections—albeit downstream of distinct signaling pathways,^73^^,^^74^ demonstrating reciprocal protection between different cell types during bacterial infection. Interestingly, enteric neurons also directly contribute to mucosal host defense. Neuronal secretion of IL-18 is required to control Salmonella infection by acting on goblet cells to enhance barrier defense, while IL-18 derived from epithelial or immune cells does not confer similar protection.^75^ These findings raise important questions about which additional cytokines neurons may produce during infection.

#### Citrobacter rodentium

*Citrobacter rodentium* infection highlights how intestinal neuronal signaling modulates innate lymphoid cell (ILC) function to shape mucosal immunity. IL-22 production by ILC3s is critical for protection against *Citrobacter*. During infection, chemogenetic activation of enteric Vasoactive Intestinal Peptide (VIP)ergic neurons decreased IL-22 production by ILC3s, resulting in increased bacterial load and decreased survival.^76^ Conversely, inhibition of VIPergic neurons was protective against bacterial spread to other organs.

### Helminth infection

Helminth infections follow a complex migratory route through multiple tissues, eliciting distinct neuroimmune interactions at each site. Infective larvae migrate to the lungs, where ILC2s expressing neuromedin U (NMU) receptor 1 (*Nmur1*) localize near NMU-expressing cholinergic neurons. NMU signaling drives ILC2 proliferation and production of IL-5, IL-13, and amphiregulin, promoting mast cell and eosinophil expansion and thereby strengthening anti-helminth immunity.^77–79^ Helminth larvae then migrate to the gut, where subsets of enteric neurons express receptors for IL-4 and IL-13; *Il13ra1* signaling regulates β-CGRP and NMU, establishing an immune–neural feedback loop regulating helminth immunity.^80^^,^^81^ This bidirectional crosstalk likely serves as a regulatory mechanism to maintain the balance between protective immunity and chronic inflammation.

In parallel, ILC2s also interact with sympathetic nerves, as they express ADRB2 and colocalize with adrenergic fibers.^12^ Adrenergic signaling through ADRB2 serves a similar regulatory function: its absence leads to increased type 2 cytokine production and reduced helminth burden in the gut.^82^ In addition, helminth infection induces cholinergic remodeling of ILC2s, which upregulate ChAT and ACh receptors.^83^ ACh administration amplifies type 2 cytokine production and helminth expulsion, whereas ChAT deficiency in ILC2s dampens anti-helminth immunity. Helminth infection models therefore illustrate how a single immune challenge can activate multiple neuroimmune communication pathways, which influence the same immune cell type. The temporal coordination of these pathways, and whether they act synergistically, remains to be determined.

Taken together, these studies reveal that peripheral nerves are integral components of immune regulation across diverse infection contexts. Through the release of neurotransmitters, neuropeptides, and cytokines, nerves actively shape immune cell differentiation, trafficking, and function. In turn, immune cells reciprocally influence neuronal survival and activity, establishing a bidirectional communication network that balances host defense and inflammation.

### Allergic response

Allergic responses are driven by the type 2 cytokines IL-4, IL-5, and IL-13, together with epithelial alarmins, and commonly arise in densely innervated barrier tissues including the airways and skin, where neuronal signals have been shown to modulate immune activity and vice versa.

#### Contact hypersensitivity and atopic dermatitis

Contact hypersensitivity (CHS) and atopic dermatitis provide 2 examples of neuroimmune circuits shaping allergic disease activity. CGRP and IL-4 both regulate CHS responses in a context-dependent manner. In a Th1-driven CHS model, CGRP suppresses dermal DC migration to lymph nodes, whereas in Th2-driven CHS, CGRP enhances IL-4 production by T cells. Thus, CGRP may inhibit type 1 but promote type 2 driven inflammation depending on the prevailing cytokine milieu.^84^ Conversely, type 2 cytokines can also directly activate murine and human sensory neurons, as demonstrated in atopic dermatitis.^85^ Stimulation of IL-4Rα sensitizes sensory neurons in the skin to pruritogens such as IL-31 and histamine, and sensory neuron-specific deletion of IL-4Rα or downstream effector JAK1 attenuates chronic itch. A distinct subset of sensory neurons coexpress IL-4Rα, IL-31Rα, TRPV1, and TRPA1, mediating the release of CGRP and SP and enabling IL-31–induced itch.^85^^,^^86^ Together, these findings link type 2 cytokines to neuronal activation and neurogenic inflammation in allergic disease.

#### House dust mites

House dust mites (HDMs) can also directly modulate sensory nerve function. During HDM-induced skin inflammation, activation of TRPV1^+^ nociceptors triggers SP release, which promotes type 2 inflammation by inducing mast cell degranulation.^87^ Notably, direct injection of SP into the skin is sufficient to elicit mast cell degranulation, leading to acute swelling and neutrophil recruitment.^88^ These observations suggest that local clusters of TRPV1^+^ neurons and mast cells may function as early neuroimmune sensors in allergic contexts. During allergic responses, DCs colocalize with sensory neurons, which relay the allergen exposure to DCs, functioning as an “allergen sensor,” and promote a type 2 immune response by driving dermal DC migration to the skin draining lymph nodes via SP.^89^

In the lung, HDM exposure similarly engages neuroimmune circuits. IL-5 acts directly on Nav1.8^+^ sensory neurons, inducing production of VIP, which in turn activates ILC2s to secrete type 2 effector cytokines and drives CD4^+^ T cell recruitment and activation.^90^ Deletion of Nav1.8^+^ neurons alleviates HDM-induced inflammation, underscoring their proinflammatory role. In allergic asthma, the neuropeptide NMU amplifies ILC2-driven allergic inflammation, and loss of NMU–NMUR1 signaling reduces ILC2 frequency and function following allergen challenge.^77^

#### Allergic lung inflammation

Recent work shows that vagal sensory neurons promote immune homeostasis in the lung through a JAK1–CGRPβ–ILC2 axis.^91^ Loss of neuronal *Jak1* exacerbates allergic inflammation and alters neuropeptide expression, including CGRPβ and SP. CGRPβ constrains type 2 cytokine production by ILC2s and limits allergic lung inflammation, and expression of a human JAK1 gain-of-function mutation suppresses inflammation. These findings highlight a potential tissue-specific therapeutic application of JAK inhibitors, and, together with the previously mentioned studies, reveal that allergic inflammation is governed by dynamic, bidirectional communication between sensory neurons and immune cells.

Across barrier tissues, emerging work on type 2 immune responses has underscored that neurotransmitters and neuropeptides can exert highly context-dependent immunomodulatory effects. This is especially evident for CGRP: In contact hypersensitivity models, CGRP has been shown to differentially impact T helper cell responses, inhibiting type 1 but promoting type 2 inflammation.^84^ In the airways, sensory neuron- and ILC2-derived CGRP can act as a brake on lung inflammation, differentially suppressing ILC2 cytokine production.^91–93^ In models of gastrointestinal inflammation, CGRP has similarly been found to have both pro- and anti-inflammatory functions.^94–96^ Future studies will be needed to better understand the nuances of this complexity.

### Immune-mediated diseases

#### Psoriasis

Neuroimmune interactions are increasingly recognized as critical modulators of autoimmune inflammation, particularly in diseases driven by the IL-23/IL-17 axis. In psoriasis, activated Th1 cells infiltrate the skin and secrete IFN-γ, which stimulates local antigen-presenting cells to produce IL-1 and IL-23, promoting the expansion and survival of IL-17–producing CD4^+^ and CD8^+^ T cells.^97^^,^^98^ Nociceptive neurons play a direct role in initiating and amplifying this type 17 inflammation, forming close contacts with IL-23–producing dermal DCs, regulating their activation and driving downstream IL-17 production by CD4^+^ T cells that mediate skin pathology.^99^ Ablation of TRPV1^+^ or Nav1.8^+^ sensory neurons reduces IL-23 production by DCs and alleviates type 17 inflammation, underscoring the neuronal contribution to disease.

Neurotrophins further link neuronal activity to immune activation in psoriasis. NGF is secreted by both sensory and sympathetic neurons in the periphery and regulates production of both CGRP and SP.^97^^,^^100^ NGF receptors are expressed on multiple immune cell types, and NGF recruits and activates T cells and mast cells at sites of psoriatic inflammation.^101^ Together, these findings demonstrate that neurons actively shape IL-23– and IL-17–driven inflammation in psoriasis, positioning neural pathways as potential therapeutic targets in autoimmune skin disease.

#### Inflammatory bowel disease

The intestine is the body’s largest barrier surface, tasked not only with nutrient absorption but also with continuous surveillance of the microbiome and maintenance of an effective defense against infection. To meet these demands, the intestinal wall is richly innervated and densely populated by immune cells, integrating the body’s two major surveillance systems. Distinctly, the intestine harbors the enteric nervous system, a specialized intrinsic network that functions largely independently of the CNS to coordinate motility, sensation, and secretion.

Building on this close anatomical integration, recent studies have revealed that intestinal immunity is tightly regulated by neuronal inputs and vice versa: neurons release classical transmitters and neuropeptides—including ACh, noradrenaline, VIP, CGRP, and SP—that act on receptors expressed by macrophages, ILCs, T cells, DCs, mast cells, and epithelial cells.^102^ In turn, immune-derived mediators modulate neuronal excitability, transmitter release, and gene expression (as reviewed in Wallrapp et al.).^103^ Acute stress enhances leukocyte recruitment to inflamed tissues,^104^ whereas chronic stress suppresses protective immunity.^105^ Histamine similarly enhances nociceptor excitability via H1 receptor–dependent sensitization of TRPV1, TRPA1, and TRPV4 channels.^106^^,^^107^ Notably, visceral pain hypersensitivity is a hallmark of inflammatory bowel disease (IBD), and mucosal supernatants from patients contain elevated histamine levels, consistent with neuronal sensitization.^108^^,^^109^ Together, these observations underscore the bidirectional crosstalk between the nervous and immune systems in the gut and motivate efforts to delineate the underlying cellular circuits and molecular mediators.

IBD encompasses a group of chronic inflammatory disorders, primarily represented by Crohn’s disease and ulcerative colitis, and substantial progress has been made in understanding how IBD pathology influences intestinal neuroimmune circuits and vice versa. Multiple, and at times conflicting, studies implicate the autonomic nervous system in IBD. Increased levels of tyrosine hydroxylase, a key enzyme in noradrenaline synthesis, have been detected in the intestine of Crohn’s disease patients,^110^ and optogenetic activation of colonic sympathetic fibers attenuates IBD disease severity in mice.^111^ This local benefit contrasts with both experimental and clinical evidence showing that systemic adrenergic activation and psychological stress exacerbate IBD.^112–114^ A plausible reconciliation of these divergent findings is receptor- and compartment-specific signaling: distinct adrenergic receptors exert nonredundant effects, their expression may vary across intestinal cell types and regions, and local versus systemic catecholamine levels may engage different targets and circuits. Beyond its immunomodulatory role, the sympathetic nervous system can also contribute to persistent visceral hypersensitivity, as chronic colitis increases catecholaminergic innervation of sensory neurons.^115^

In addition to sympathetic regulation, the parasympathetic branch of the autonomic nervous system also plays a pivotal role in intestinal inflammation. Studies have reported reduced vagal tone in IBD, and clinical data suggest that vagus nerve stimulation (VNS) can ameliorate disease activity,^116^ supporting neuromodulation as a therapeutic approach. Consistent with this, mouse models demonstrate that central cholinergic activation via acetylcholinesterase inhibition attenuates mucosal inflammation,^117^ whereas vagotomy exacerbates colitis.^118^^,^^119^

Further, cholinergic enteric neurons sense mechanical force via Piezo1 to regulate intestinal motility and maintain immune homeostasis.^120^ Enteric mechanosensation mediated by Piezo1 in neurons controls ACh release and prevents aberrant inflammation and tissue damage in mouse models of IBD.

Sensory nerves also influence disease severity in IBD. Chemogenetic activation of TRPV1^+^ nociceptors is protective against colitis, whereas neuronal ablation or silencing exacerbates disease.^121^^,^^122^ Consistent with a homeostatic role, intestinal biopsies from patients with IBD show dysregulated nociceptor gene expression.^121^ Mechanistically, sensory fibers release SP and CGRP, although their effects are context dependent. In dextran sulfate sodium colitis, SP deficiency phenocopies TRPV1^+^ nerve silencing, increasing disease susceptibility,^121^ whereas in oxazolone colitis, SP-deficient mice are protected, and CGRP-deficient mice are more susceptible.^95^ Similarly, VIP exhibits both pro- and anti-inflammatory effects in TNBS colitis models.^123^^,^^124^ In aggregate, these findings illustrate that neuroimmune regulation in IBD is profoundly context dependent—varying by colitis model, nerve type, neurotransmitter, and receptor expression—and underscore the need for additional mechanistic studies that resolve this complexity.

While the nervous system is now recognized as a critical regulator of intestinal immunity in general, and IBD pathology in particular, our understanding of its full complexity remains limited. Multiple neuronal lineages, many neurotransmitters and neuropeptides, and cell type–specific receptor repertoires together generate combinatorial signaling networks. Moreover, inflammation and infection dynamically rewire both immune programs and neuronal transcriptomes, offering new opportunities to uncover the principles governing neuroimmune circuits.

## Clinical and translational implications

Advances in our understanding of neuroimmune crosstalk have driven both the creation of new therapeutics and the repurposing of existing drugs to target these pathways. These treatments range from agents that block nerve-to-immune signals (e.g. blocking neurotransmitter receptors on immune cells), to antibodies that block immune-to-nerve signals (e.g. cytokine receptors associated with itch) as well as bioelectronic interventions. Each exemplifies how intervening in the neural-immune conversation can translate to clinical benefit.

Perhaps one of the most promising areas for clinical translation of neuroimmune research is oncology. Solid tumors are now understood to not only be infiltrated by immune cells but also innervated by multiple types of nerves, which functionally regulate the TME. The identification of relevant receptor-ligand interactions has sparked a wave of clinical investigation to determine which drugs targeting these pathways have clinical benefits for cancer patients, specifically also when combined with classical immune checkpoint blockade (ICB). Importantly, many drugs targeting neurotransmitter or neuropeptide receptors are already pharmacologically well characterized due to their prior clinical use in other diseases, providing established safety profiles that could accelerate their repurposing for anticancer therapy.^29^^,^^125^

One of the most explored drug types in this context are beta-blockers, which selectively or unselectively block beta-adrenergic receptors expressed on immune and nonimmune cells, including cancer cells. Preclinical evidence demonstrates that adrenergic signaling profoundly shapes immune cell function and tumor progression,^2^^,^^13^^,^^34^ providing the rationale for testing beta-blockers as adjuncts to immunotherapy. However, current clinical evidence of their efficacy remains inconsistent across cancer types and treatment settings, likely in part due to retrospective study design.^13^ Retrospective studies and meta-analyses show promising results for beta-blockers in melanoma, non-small cell lung cancer, and urothelial carcinoma, and results from other studies suggest that a subgroup of patients may derive benefit from beta-blocker use in combination with ICB, although we are currently lacking the predictive biomarkers necessary to reliably identify this population.^13^^,^^126–128^ Besides the limitations of heterogeneous patient cohorts and tumor types, beta-blocker use clinically often selects for older patients, underscoring the need for prospective trials to mitigate this bias, many of which are currently ongoing.

In addition to beta-blockers, selective serotonin reuptake inhibitors, widely prescribed antidepressants, have recently demonstrated antitumor activity in multiple preclinical models and shown therapeutic synergy with ICB,^129^ highlighting the potential to leverage other neuroimmune axes therapeutically. Similarly, sensory nerve–derived neuropeptides such as CGRP and SP are emerging as promising therapeutic targets, and preclinical studies report antitumor effects of SP receptor antagonists, including the clinically approved antiemetic aprepitant.^28^ Together, these findings indicate that signaling pathways once considered exclusive to the nervous system may represent novel therapeutic targets within the immune system.

Conversely, several clinically approved drugs modulate neuroimmune communication by suppressing immune-derived mediators that act on neurons. Dupilumab, a monoclonal antibody against IL-4Rα, blocks IL-4 and IL-13 signaling at the immune–sensory nerve interface, thereby attenuating type 2–mediated pathologies including allergic inflammation and chronic itch.^85^ Similarly, upadacitinib, a JAK1 inhibitor, blocks cytokine receptor signaling that activates or sensitizes peripheral neurons, improving skin inflammation and providing relief from itch in atopic dermatitis.^85^ Thus, signaling pathways traditionally attributed to the nervous system may represent new therapeutic targets within the immune system and vice versa, opening new therapeutic opportunities across both systems.

Beyond pharmacologic strategies, bioelectronic interventions are showing promise in chronic inflammatory diseases like IBD and rheumatoid arthritis: VNS engages the cholinergic anti-inflammatory pathway,^6^^,^^130–134^ leading to acetylcholine release that acts on macrophages and other immune cells to suppress proinflammatory cytokine production. Early clinical trials have demonstrated its potential in IBD, in which VNS reduced intestinal inflammation, and in rheumatoid arthritis, in which it lowered TNF levels and disease activity.^103^^,^^116^^,^^117^^,^^135^^,^^136^

Together, these treatment strategies underscore the impressive translational potential of targeting neuroimmune interactions. It is now clear that modulating the neuroimmune dialogue—whether through repurposed drugs such as antihypertensives like beta-blockers and antidepressants like selective serotonin reuptake inhibitors, cytokine-directed biologics like dupilumab, small-molecule inhibitors like upadacitinib, or bioelectronic approaches like VNS—can yield profound clinical benefit. As mechanistic insights continue to emerge, the deliberate targeting of neuroimmune pathways holds promise not only for refractory inflammatory and allergic diseases, but also as a powerful new frontier in cancer immunotherapy.

## On the horizon for peripheral neuroimmunology

### Current limitations

Despite the rapid growth of (peripheral) neuroimmunology, it is a young field, and our understanding of the neuroimmune connectome remains fragmented. Immune cells (and many other cell types) express a broad repertoire of receptors for neurotransmitters and neuropeptides, but a systematic characterization of peripheral cells responsive to neural signals is still lacking. An integrated view of this expression and its dynamics across physiological and pathological contexts is needed to more comprehensively define neuroimmune regulation. This must be complemented by models allowing for nerve or immune cell type specific perturbations such as Cre/lox systems, to distinguish direct and indirect effects of neuronal signaling. Large-scale CRISPR-based screens across multiple nerve types in shared tissue contexts will enable systematic mapping of dominant neuroimmune circuits.

As a new field, neuroimmunology also faces significant technological limitations. Progress depends on expanding and adapting the methodological repertoire of both neuroscience and immunology for use in human and mouse samples. For example, multiparametric single-cell analyses like flow cytometry, commonly used in immunology, are hampered by limited availability of reliable antibodies for neurotransmitter and neuropeptide targets.

Despite a fundamental understanding that spatial proximity between neurons and immune cells is critical for their interaction, it remains challenging to characterize neuroimmune hubs in intact tissues. Visualizing neurotransmitters and neuropeptides with conventional imaging approaches is difficult, and standard spatial transcriptomics technologies are limited by thin section thickness, restricting our ability to fully capture inherently 3-dimensional structures like nerves, and to comprehensively characterize the cell types in perineural niches. When neuronal cell bodies reside outside the tissue of interest, the sparsity of transcripts in their processes poses an additional challenge for accurate molecular profiling. Improved methods integrating transcriptomic data with multiplexed immunofluorescence and/or complementary viral labeling approaches are needed, and recent advances in computational modeling, and spatial multiomics may help to address this.

It will also be important to gain more precise temporal and spatial control over neuronal activation or silencing. While neuroscience is far ahead of immunology in its use of optogenetic and chemogenetic approaches, stimulation or silencing regimes require adaptation to immunological experimental systems, in which time scales are often drastically longer, viral gene delivery may confound immune phenotypes, and stimulation probes cannot be anchored in solid bone structures. Refining these approaches will help elucidate the temporal regulation of immune outcomes. Moreover, because neuronal signaling occurs throughout the body, it will be critical to develop strategies that enable tissue-specific targeting of these pathways to dissect their local functions.

### Outlook

Neuroimmunology is not merely a discipline, but also a conceptual framework for understanding physiological networks. It is an inherently nonreductionist perspective that integrates multicellular and multiorgan communication, environmental cues, and behavioral states. Moving beyond isolated observations, the field now requires coordinated initiatives—shared atlases, standardized perturbation datasets, and cross-disciplinary collaborations—to comprehensively chart the neuroimmune connectome.

For peripheral neuroimmunology to achieve lasting impact, it must link mechanistic insight to human disease modulation, much like the advent of immune checkpoint therapy has catalyzed progress in tumor immunology. True innovation will be measured not only by scientific novelty, but also by translation. From chronic itch to infection and cancer, the neuroimmune interface represents a fertile ground for therapeutic intervention. The next decade will hinge on our capacity to adapt technologies and refine models to improve our ability to “listen in” on the molecular dialogue between nerves and immune cells and gain insights that go beyond classically appreciated roles for each system.

Conceptually, these findings position the nervous system as a novel “state setter” of immunity that shapes T cell fate and spatial dynamics. Understanding how distinct neuronal pathways influence T cell activation, differentiation, function, and migration has high translational potential, opening new avenues for neuromodulation as a form of precision immunotherapy. By therapeutically targeting neuroimmune communication, it may become possible to fine-tune immune responses and bias immune cells toward specific differentiation states—enhancing protective antiviral and antitumor immunity while restraining overactive immune responses.

Nerves as “state-setters” of immunity In response to an antigen challenge, T cells acquire a broad spectrum of differentiation states, generating distinct T cell subsets with specialized phenotypic, functional, and migratory properties. This heterogeneity is essential for context-dependent pathogen control and the generation of long-lived protective immunity. In general, naïve T cells follow 2 principal differentiation trajectories, that culminate in the formation of either memory or exhausted T cell populations, with trajectory branches generating shorter-lived effector populations. These developmental pathways are orchestrated by 4 key signals: antigen, costimulation, cytokines, and nutrients.^137–139^

Emerging evidence now suggests that neuronal inputs represent an additional regulatory layer shaping T cell differentiation and function. Activation of sensory nerves alone, even in the absence of infection, is sufficient to induce IL-23 production by DCs, which in turn drives differentiation of Th17 cells.^69^^,^^140^ Sensory nerves also promote antiviral effector T cell responses to HSV, as evidenced by the impaired CD8 T cell response in nociceptor-deficient mice.^60^ During acute viral infection, CGRP signaling through RAMP3 promotes Th1 differentiation and effector cytokine production while concurrently inhibiting Th2 polarization.^65^ Interestingly, CGRP effects appear context dependent, as it instead suppresses Th1 differentiation and enhances Th2 polarization in the context of contact hypersensitivity.^84^ In chronic viral infection, sympathetic nerves release noradrenaline, which acts on the beta1 adrenergic receptor expressed by CD8^+^ T cells, promoting their progression toward terminal effector and exhausted states.^2^ Collectively, these findings demonstrate that distinct neuronal pathways dynamically regulate T cell differentiation trajectories across infection contexts.

Beyond modulating T cell differentiation, nerves also govern immune cell trafficking across tissues, a critical function of effector cells.^52^ During viral infection, sympathetic nerve activation inhibits lymphocyte migration within tissues and dampens T cell responses.^141^ Future work will be required to determine whether specific T cell subsets—naïve, effector, memory, or exhausted—can be differentially directed to certain organs or tissue niches in response to neuronal activation. Such mechanisms could, for example, promote immune priming of naïve cells in lymphoid tissues or direct antigen-specific effector cells to peripheral tissues for rapid pathogen clearance.

## References

[1] Godinho-Silva C , Cardoso F, Veiga-Fernandes H. Neuro-immune cell units: a new paradigm in physiology. Annu Rev Immunol. 2019;37: 19–46. 30379595 10.1146/annurev-immunol-042718-041812

[2] Globig A-M et al The beta(1)-adrenergic receptor links sympathetic nerves to T cell exhaustion. Nature. 2023;622:383–392. 37731001 10.1038/s41586-023-06568-6 PMC10871066

[3] Kolter J et al Sensory neurons shape local macrophage identity via TGF-beta signaling. Immunity. 2025;58:2556–2573.e8., 40914152 10.1016/j.immuni.2025.08.004

[4] Pirzgalska RM et al Sympathetic neuron-associated macrophages contribute to obesity by importing and metabolizing norepinephrine. Nat Med. 2017;23:1309–1318. 29035364 10.1038/nm.4422 PMC7104364

[5] Stead RH et al Intestinal mucosal mast cells in normal and nematode-infected rat intestines are in intimate contact with peptidergic nerves. Proc Natl Acad Sci U S A. 1987;84:2975–2979. 2437589 10.1073/pnas.84.9.2975 PMC304783

[6] Tracey KJ. The inflammatory reflex. Nature. 2002;420:853–859. 12490958 10.1038/nature01321

[7] Schiller M , Ben-Shaanan TL, Rolls A. Neuronal regulation of immunity: why, how and where? Nat Rev Immunol. 2021;21:20–36. 32811994 10.1038/s41577-020-0387-1

[8] Koren T et al Insular cortex neurons encode and retrieve specific immune responses. Cell. 2021;184:5902–5915 e5917. 34752731 10.1016/j.cell.2021.10.013

[9] Kim BS , Artis D. The sensory neuroimmune frontier. Immunity. 2025;58:1033–1039. 40324378 10.1016/j.immuni.2025.03.018

[10] Chu C , Artis D, Chiu IM. Neuro-immune Interactions in the Tissues. Immunity. 2020;52:464–474. 32187517 10.1016/j.immuni.2020.02.017 PMC10710744

[11] Huh JR , Veiga-Fernandes H. Neuroimmune circuits in inter-organ communication. Nat Rev Immunol. 2020;20:217–228. 31848462 10.1038/s41577-019-0247-z

[12] Klein Wolterink RGJ , Wu GS, Chiu IM, Veiga-Fernandes H. Neuroimmune interactions in peripheral organs. Annu Rev Neurosci. 2022;45:339–360. 35363534 10.1146/annurev-neuro-111020-105359 PMC9436268

[13] Amit M et al Neuro-immune cross-talk in cancer. Nat Rev Cancer. 2025;25:573–589. 40523971 10.1038/s41568-025-00831-w PMC13142818

[14] Chen G et al PD-L1 inhibits acute and chronic pain by suppressing nociceptive neuron activity via PD-1. Nat Neurosci. 2017;20:917–926. 28530662 10.1038/nn.4571 PMC5831162

[15] Ernst P. Ueber das Wachstum und die Verbreitung bosartiger Geschwulste, insbesondere des Krebses in den Lymphbahnen der Nerven; ein Beitrag zur Biologie des Krebses [On the growth and spread of malignant tumors, especially cancer in the lymphatic vessels of the nerves; a contribution to the biology of cancer]. Beitr Anat Path. 1905;7:29–51.

[16] De Sousa Pereira A. A basis for sympathectomy for cancer of the cervix uteri. Arch Surg (1920). 1946;52:260–285. 21024600 10.1001/archsurg.1946.01230050265003

[17] Young HH. On the presence of nerves in tumors and of other structures in them as revealed by a modification of Ehrlich’s method of “Vital Staining” with methylene blue. J Exp Med. 1897;2:1–12. 10.1084/jem.2.1.1 PMC2117917 19866822

[18] Oertel H. Innervation and tumour growth: a preliminary report. Can Med Assoc J. 1928;18:135–139. 20316697 PMC1709538

[19] Hanahan D , Weinberg RA. Hallmarks of cancer: the next generation. Cell. 2011;144:646–674. 21376230 10.1016/j.cell.2011.02.013

[20] Albo D et al Neurogenesis in colorectal cancer is a marker of aggressive tumor behavior and poor outcomes. Cancer. 2011;117:4834–4845. 21480205 10.1002/cncr.26117

[21] Liebl F et al The severity of neural invasion is associated with shortened survival in colon cancer. Clin Cancer Res. 2013;19:50–61. 23147996 10.1158/1078-0432.CCR-12-2392

[22] Reavis HD , Chen HI, Drapkin R. Tumor innervation: cancer has some nerve. Trends Cancer. 2020;6:1059–1067. 32807693 10.1016/j.trecan.2020.07.005 PMC7688507

[23] Magnon C et al Autonomic nerve development contributes to prostate cancer progression. Science. 2013;341:1236361. 23846904 10.1126/science.1236361

[24] Zahalka AH et al Adrenergic nerves activate an angio-metabolic switch in prostate cancer. Science. 2017;358:321–326. 29051371 10.1126/science.aah5072 PMC5783182

[25] Amit M et al Loss of p53 drives neuron reprogramming in head and neck cancer. Nature. 2020;578:449–454. 32051587 10.1038/s41586-020-1996-3 PMC9723538

[26] Saloman JL et al Ablation of sensory neurons in a genetic model of pancreatic ductal adenocarcinoma slows initiation and progression of cancer. Proc Natl Acad Sci U S A. 2016;113:3078–3083. 26929329 10.1073/pnas.1512603113 PMC4801275

[27] Zhi X et al Nociceptive neurons promote gastric tumour progression via a CGRP-RAMP1 axis. Nature. 2025;640:802–810. 39972142 10.1038/s41586-025-08591-1 PMC13022952

[28] Padmanaban V et al Neuronal substance P drives metastasis through an extracellular RNA-TLR7 axis. Nature. 2024;633:207–215. 39112700 10.1038/s41586-024-07767-5 PMC11633843

[29] Yaniv D , Mattson B, Talbot S, Gleber-Netto FO, Amit M. Targeting the peripheral neural-tumour microenvironment for cancer therapy. Nat Rev Drug Discov. 2024;23:780–796. 39242781 10.1038/s41573-024-01017-z PMC12123372

[30] Zhang Y et al Cancer cells co-opt nociceptive nerves to thrive in nutrient-poor environments and upon nutrient-starvation therapies. Cell Metab. 2022;34:1999–2017 e1910. 36395769 10.1016/j.cmet.2022.10.012

[31] Hanahan D , Michielin O, Pittet MJ. Convergent inducers and effectors of T cell paralysis in the tumour microenvironment. Nat Rev Cancer. 2025;25:41–58. 39448877 10.1038/s41568-024-00761-z

[32] De Palma M , Hanahan D. Milestones in tumor vascularization and its therapeutic targeting. Nat Cancer. 2024;5:827–843. 38918437 10.1038/s43018-024-00780-7

[33] Chi H , Pepper M, Thomas PG. Principles and therapeutic applications of adaptive immunity. Cell. 2024;187:2052–2078. 38670065 10.1016/j.cell.2024.03.037 PMC11177542

[34] Mohammadpour H et al beta2 adrenergic receptor-mediated signaling regulates the immunosuppressive potential of myeloid-derived suppressor cells. J Clin Invest. 2019;129:5537–5552. 31566578 10.1172/JCI129502 PMC6877316

[35] Nakai A , Hayano Y, Furuta F, Noda M, Suzuki K. Control of lymphocyte egress from lymph nodes through beta2-adrenergic receptors. J Exp Med. 2014;211:2583–2598. 25422496 10.1084/jem.20141132 PMC4267238

[36] Qiao G et al beta-Adrenergic signaling blocks murine CD8(+) T-cell metabolic reprogramming during activation: a mechanism for immunosuppression by adrenergic stress. Cancer Immunol Immunother. 2019;68:11–22. 30229289 10.1007/s00262-018-2243-8 PMC6326964

[37] Balood M et al Nociceptor neurons affect cancer immunosurveillance. Nature. 2022;611:405–412. 36323780 10.1038/s41586-022-05374-w PMC9646485

[38] Zheng C et al Tumor-specific cholinergic CD4(+) T lymphocytes guide immunosurveillance of hepatocellular carcinoma. Nat Cancer. 2023;4:1437–1454. 37640929 10.1038/s43018-023-00624-w PMC10597839

[39] Zheng C , Liu S, Fazel Modares N, St Paul M, Mak TW. Cholinergic T cells revitalize the tumor immune microenvironment: TIME to ChAT. Nat Immunol. 2025;26:665–677. 40307453 10.1038/s41590-025-02144-4

[40] Zhang B et al B cell-derived GABA elicits IL-10(+) macrophages to limit anti-tumour immunity. Nature. 2021;599:471–476. 34732892 10.1038/s41586-021-04082-1 PMC8599023

[41] Schneider MA et al Attenuation of peripheral serotonin inhibits tumor growth and enhances immune checkpoint blockade therapy in murine tumor models. Sci Translational Med. 2021;13:eabc8188. 10.1126/scitranslmed.abc8188 34524861

[42] Jurgens HA , Amancherla K, Johnson RW. Influenza infection induces neuroinflammation, alters hippocampal neuron morphology, and impairs cognition in adult mice. J Neurosci. 2012;32:3958–3968. 22442063 10.1523/JNEUROSCI.6389-11.2012 PMC3353809

[43] Reel JM , Abbadi J, Cox MA. T cells at the interface of neuroimmune communication. J Allergy Clin Immunol. 2024;153:894–903. 37952833 10.1016/j.jaci.2023.10.026 PMC10999355

[44] Poller WC et al Brain motor and fear circuits regulate leukocytes during acute stress. Nature. 2022;607:578–584. 35636458 10.1038/s41586-022-04890-z PMC9798885

[45] Estrada LD , Ağaç D, Farrar JD. Sympathetic neural signaling via the β2‐adrenergic receptor suppresses T‐cell receptor‐mediated human and mouse CD8^+^ T‐cell effector function. Eur J Immunol. 2016;46:1948–1958. 27222010 10.1002/eji.201646395 PMC5241047

[46] Grebe KM et al Cutting edge: sympathetic nervous system increases proinflammatory cytokines and exacerbates influenza A virus pathogenesis. J Immunol. 2010;184:540–544. 20018617 10.4049/jimmunol.0903395 PMC2941093

[47] Grebe KM et al Sympathetic nervous system control of anti-influenza CD8+ T cell responses. Proc Natl Acad Sci U S A. 2009;106:5300–5305. 19286971 10.1073/pnas.0808851106 PMC2664017

[48] Dhabhar FS , Viswanathan K. Short-term stress experienced at time of immunization induces a long-lasting increase in immunologic memory. Am J Physiol Regul Integr Comp Physiol. 2005;289:R738–744. 15890793 10.1152/ajpregu.00145.2005

[49] Padgett DA , Glaser R. How stress influences the immune response. Trends Immunol. 2003;24:444–448. 12909458 10.1016/s1471-4906(03)00173-x

[50] Bucsek MJ , Giridharan T, MacDonald CR, Hylander BL, Repasky EA. An overview of the role of sympathetic regulation of immune responses in infectious disease and autoimmunity. Int J Hyperthermia. 2018;34:135–143. 29498310 10.1080/02656736.2017.1411621 PMC6309867

[51] Coutinho AE , Chapman KE. The anti-inflammatory and immunosuppressive effects of glucocorticoids, recent developments and mechanistic insights. Mol Cell Endocrinol. 2011;335:2–13. 20398732 10.1016/j.mce.2010.04.005 PMC3047790

[52] Mueller SN. Neural control of immune cell trafficking. J Exp Med. 2022;219:e20211604. 10.1084/jem.20211604 PMC8932541 35195682

[53] Almanzar N et al Vagal TRPV1(+) sensory neurons protect against influenza virus infection by regulating lung myeloid cell dynamics. Sci Immunol. 2025;10:eads6243.40749036 10.1126/sciimmunol.ads6243

[54] Cembellin-Prieto A , Luo Z, Kulaga H, Baumgarth N. B cells modulate lung antiviral inflammatory responses via the neurotransmitter acetylcholine. Nat Immunol. 2025;26:775–789. 40263611 10.1038/s41590-025-02124-8 PMC12043518

[55] Baral P , Udit S, Chiu IM. Pain and immunity: implications for host defence. Nat Rev Immunol. 2019;19:433–447. 30874629 10.1038/s41577-019-0147-2 PMC6700742

[56] Fields HL , Rowbotham M, Baron R. Postherpetic neuralgia: irritable nociceptors and deafferentation. Neurobiol Dis. 1998;5:209–227. 9848092 10.1006/nbdi.1998.0204

[57] Steiner I , Kennedy PG, Pachner AR. The neurotropic herpes viruses: herpes simplex and varicella-zoster. Lancet Neurol. 2007;6:1015–1028. 17945155 10.1016/S1474-4422(07)70267-3

[58] Khanna KM , Bonneau RH, Kinchington PR, Hendricks RL. Herpes simplex virus-specific memory CD8+ T cells are selectively activated and retained in latently infected sensory ganglia. Immunity. 2003;18:593–603. 12753737 10.1016/s1074-7613(03)00112-2 PMC2871305

[59] Freeman ML , Sheridan BS, Bonneau RH, Hendricks RL. Psychological stress compromises CD8+ T cell control of latent herpes simplex virus type 1 infections. J Immunol. 2007;179:322–328. 17579052 10.4049/jimmunol.179.1.322 PMC2367250

[60] Filtjens J et al Nociceptive sensory neurons promote CD8 T cell responses to HSV-1 infection. Nat Commun. 2021;12:2936. 34006861 10.1038/s41467-021-22841-6 PMC8131384

[61] McGavern DB , Homann D, Oldstone MB. T cells in the central nervous system: the delicate balance between viral clearance and disease. J Infect Dis. 2002;186 Suppl 2:S145–151. 12424690 10.1086/344264 PMC5319418

[62] Urban SL et al Peripherally induced brain tissue–resident memory CD8+ T cells mediate protection against CNS infection. Nat Immunol. 2020;21:938–949. 32572242 10.1038/s41590-020-0711-8 PMC7381383

[63] Rosato PC et al Tissue-resident memory T cells trigger rapid exudation and local antibody accumulation. Mucosal Immunol. 2023;16:17–26. 36657662 10.1016/j.mucimm.2022.11.004 PMC10338064

[64] Ning J et al Functional virus-specific memory T cells survey glioblastoma. Cancer Immunol Immunother. 2022;71:1863–1875. 35001153 10.1007/s00262-021-03125-w PMC9271132

[65] Hou Y et al Neuropeptide signalling orchestrates T cell differentiation. Nature. 2024;635:444–452. 39415015 10.1038/s41586-024-08049-w PMC11951087

[66] Tarnawski L et al Cholinergic regulation of vascular endothelial function by human ChAT(+) T cells. Proc Natl Acad Sci U S A. 2023;120:e2212476120. 36989306 10.1073/pnas.2212476120 PMC10083572

[67] Malin SG , Shavva VS, Tarnawski L, Olofsson PS. Functions of acetylcholine-producing lymphocytes in immunobiology. Curr Opin Neurobiol. 2020;62:115–121. 32126362 10.1016/j.conb.2020.01.017

[68] Cox MA et al Choline acetyltransferase-expressing T cells are required to control chronic viral infection. Science. 2019;363:639–644. 30733420 10.1126/science.aau9072 PMC7181845

[69] Cohen JA et al Cutaneous TRPV1(+) neurons trigger protective innate type 17 anticipatory immunity. Cell. 2019;178:919–932 e914. 31353219 10.1016/j.cell.2019.06.022 PMC6788801

[70] Chiu IM et al Bacteria activate sensory neurons that modulate pain and inflammation. Nature. 2013;501:52–57. 23965627 10.1038/nature12479 PMC3773968

[71] Baral P et al Nociceptor sensory neurons suppress neutrophil and gammadelta T cell responses in bacterial lung infections and lethal pneumonia. Nat Med. 2018;24:417–426. 29505031 10.1038/nm.4501 PMC6263165

[72] Gabanyi I et al Neuro-immune interactions drive tissue programming in intestinal macrophages. Cell. 2016;164:378–391. 26777404 10.1016/j.cell.2015.12.023 PMC4733406

[73] Matheis F et al Adrenergic signaling in muscularis macrophages limits infection-induced neuronal loss. Cell. 2020;180:64–78 e16. 31923400 10.1016/j.cell.2019.12.002 PMC7271821

[74] Ahrends T et al Enteric pathogens induce tissue tolerance and prevent neuronal loss from subsequent infections. Cell. 2021;184:5715–5727 e5712. 34717799 10.1016/j.cell.2021.10.004 PMC8595755

[75] Jarret A et al Enteric nervous system-derived IL-18 orchestrates mucosal barrier immunity. Cell. 2020;180:813–814. 32084342 10.1016/j.cell.2020.02.004

[76] Talbot J et al Feeding-dependent VIP neuron-ILC3 circuit regulates the intestinal barrier. Nature. 2020;579:575–580. 32050257 10.1038/s41586-020-2039-9 PMC7135938

[77] Wallrapp A et al The neuropeptide NMU amplifies ILC2-driven allergic lung inflammation. Nature. 2017;549:351–356. 28902842 10.1038/nature24029 PMC5746044

[78] Klose CSN et al The neuropeptide neuromedin U stimulates innate lymphoid cells and type 2 inflammation. Nature. 2017;549:282–286. 28869965 10.1038/nature23676 PMC6066372

[79] Cardoso V et al Neuronal regulation of type 2 innate lymphoid cells via neuromedin U. Nature. 2017;549:277–281. 28869974 10.1038/nature23469 PMC5714273

[80] Barilla RM et al Type 2 cytokines act on enteric sensory neurons to regulate neuropeptide-driven host defense. Science. 2025;389:260–267. 40403128 10.1126/science.adn9850 PMC12632183

[81] Wang Y et al Bi-directional communication between intrinsic enteric neurons and ILC2s inhibits host defense against helminth infection. Immunity. 2025;58:465–480 e468. 39889704 10.1016/j.immuni.2025.01.004

[82] Moriyama S et al beta(2)-adrenergic receptor-mediated negative regulation of group 2 innate lymphoid cell responses. Science. 2018;359:1056–1061. 29496881 10.1126/science.aan4829

[83] Chu C et al The ChAT-acetylcholine pathway promotes group 2 innate lymphoid cell responses and anti-helminth immunity. Sci Immunol. 2021;6:eabe3218. 10.1126/sciimmunol.abe3218 PMC8577047 33674322

[84] Mikami N et al Calcitonin gene-related peptide is an important regulator of cutaneous immunity: effect on dendritic cell and T cell functions. J Immunol. 2011;186:6886–6893. 21551361 10.4049/jimmunol.1100028

[85] Oetjen LK et al Sensory neurons co-opt classical immune signaling pathways to mediate chronic itch. Cell. 2017;171:217–228 e213. 28890086 10.1016/j.cell.2017.08.006 PMC5658016

[86] Kim B et al Neuroimmune interplay during type 2 inflammation: Symptoms, mechanisms, and therapeutic targets in atopic diseases. J Allergy Clin Immunol. 2024;153:879–893. 37634890 10.1016/j.jaci.2023.08.017 PMC11215634

[87] Serhan N et al House dust mites activate nociceptor–mast cell clusters to drive type 2 skin inflammation. Nat Immunol. 2019;20:1435–1443. 31591569 10.1038/s41590-019-0493-z PMC6858877

[88] Green DP , Limjunyawong N, Gour N, Pundir P, Dong X. A mast-cell-specific receptor mediates neurogenic inflammation and pain. Neuron. 2019;101:412–420 e413. 30686732 10.1016/j.neuron.2019.01.012 PMC6462816

[89] Perner C et al Substance P release by sensory neurons triggers dendritic cell migration and initiates the type-2 immune response to allergens. Immunity. 2020;53:1063–1077 e1067. 33098765 10.1016/j.immuni.2020.10.001 PMC7677179

[90] Talbot S et al Silencing nociceptor neurons reduces allergic airway inflammation. Neuron. 2015;87:341–354. 26119026 10.1016/j.neuron.2015.06.007 PMC4506220

[91] Tamari M et al Sensory neurons promote immune homeostasis in the lung. Cell. 2024;187:44–61.e17. 38134932 10.1016/j.cell.2023.11.027 PMC10811756

[92] Wallrapp A et al Calcitonin gene-related peptide negatively regulates alarmin-driven type 2 innate lymphoid cell responses. Immunity. 2019;51:709–723.e6. 31604686 10.1016/j.immuni.2019.09.005 PMC7076585

[93] Nagashima H et al Neuropeptide CGRP limits group 2 innate lymphoid cell responses and constrains type 2 inflammation. Immunity. 2019;51:682–695.e6. 31353223 10.1016/j.immuni.2019.06.009 PMC6801073

[94] Xu H et al Transcriptional atlas of intestinal immune cells reveals that neuropeptide alpha-CGRP modulates group 2 innate lymphoid cell responses. Immunity. 2019;51:696–708.e9. 31618654 10.1016/j.immuni.2019.09.004 PMC6991097

[95] Engel MA , Becker C, Reeh PW, Neurath MF. Role of sensory neurons in colitis: increasing evidence for a neuroimmune link in the gut. Inflamm Bowel Dis. 2011;17:1030–1033. 20722067 10.1002/ibd.21422

[96] Reinshagen M et al Calcitonin gene-related peptide mediates the protective effect of sensory nerves in a model of colonic injury. J Pharmacol Exp Ther. 1998;286:657–661. 9694917

[97] Ayasse MT , Buddenkotte J, Alam M, Steinhoff M. Role of neuroimmune circuits and pruritus in psoriasis. Exp Dermatol. 2020;29:414–426. 31954075 10.1111/exd.14071

[98] Kryczek I et al Induction of IL-17+ T cell trafficking and development by IFN-gamma: mechanism and pathological relevance in psoriasis. J Immunol. 2008;181:4733–4741. 18802076 10.4049/jimmunol.181.7.4733 PMC2677162

[99] Riol-Blanco L et al Nociceptive sensory neurons drive interleukin-23-mediated psoriasiform skin inflammation. Nature. 2014;510:157–161. 24759321 10.1038/nature13199 PMC4127885

[100] Minnone G , De Benedetti F, Bracci-Laudiero L. NGF and its receptors in the regulation of inflammatory response. Int J Mol Sci. 2017;18:1028. 28492466 10.3390/ijms18051028 PMC5454940

[101] Saraceno R , Kleyn CE, Terenghi G, Griffiths CE. The role of neuropeptides in psoriasis. Br J Dermatol. 2006;155:876–882. 17034513 10.1111/j.1365-2133.2006.07518.x

[102] Zhu Y et al A chemogenetic screen reveals that Trpv1-expressing neurons control regulatory T cells in the gut. Science. 2024;385:eadk1679. 39088603 10.1126/science.adk1679 PMC11416019

[103] Wallrapp A , Chiu IM. Neuroimmune interactions in the intestine. Annu Rev Immunol. 2024;42:489–519. 38941607 10.1146/annurev-immunol-101921-042929 PMC13058849

[104] Viswanathan K , Dhabhar FS. Stress-induced enhancement of leukocyte trafficking into sites of surgery or immune activation. Proc Natl Acad Sci U S A. 2005;102:5808–5813. 15817686 10.1073/pnas.0501650102 PMC556309

[105] Dhabhar FS. Acute stress enhances while chronic stress suppresses skin immunity. The role of stress hormones and leukocyte trafficking. Ann N Y Acad Sci. 2000;917:876–893. 11268419 10.1111/j.1749-6632.2000.tb05454.x

[106] Aguilera-Lizarraga J , Hussein H, Boeckxstaens GE. Immune activation in irritable bowel syndrome: what is the evidence? Nat Rev Immunol. 2022;22:674–686. 35296814 10.1038/s41577-022-00700-9

[107] Wouters MM et al Histamine receptor H1-mediated sensitization of TRPV1 mediates visceral hypersensitivity and symptoms in patients with irritable bowel syndrome. Gastroenterology. 2016;150:875–887.e9. 26752109 10.1053/j.gastro.2015.12.034

[108] Barbara G et al Mast cell-dependent excitation of visceral-nociceptive sensory neurons in irritable bowel syndrome. Gastroenterology. 2007;132:26–37. 17241857 10.1053/j.gastro.2006.11.039

[109] Buhner S et al Activation of human enteric neurons by supernatants of colonic biopsy specimens from patients with irritable bowel syndrome. Gastroenterology. 2009;137:1425–1434. 19596012 10.1053/j.gastro.2009.07.005

[110] Belai A , Boulos PB, Robson T, Burnstock G. Neurochemical coding in the small intestine of patients with Crohn’s disease. Gut. 1997;40:767–774. 9245931 10.1136/gut.40.6.767 PMC1027202

[111] Schiller M et al Optogenetic activation of local colonic sympathetic innervations attenuates colitis by limiting immune cell extravasation. Immunity. 2021;54:1022–1036.e8. 33932356 10.1016/j.immuni.2021.04.007 PMC8116309

[112] Bai A , Lu N, Guo Y, Chen J, Liu Z. Modulation of inflammatory response via alpha2-adrenoceptor blockade in acute murine colitis. Clin Exp Immunol. 2009;156:353–362. 19250273 10.1111/j.1365-2249.2009.03894.x PMC2759485

[113] Zádori ZS et al Inhibition of alpha2A-adrenoceptors ameliorates dextran sulfate sodium-induced acute intestinal inflammation in mice. J Pharmacol Exp Ther. 2016;358:483–491. 27418171 10.1124/jpet.116.235101

[114] Schneider KM et al The enteric nervous system relays psychological stress to intestinal inflammation. Cell. 2023;186:2823–2838.e20. 37236193 10.1016/j.cell.2023.05.001 PMC10330875

[115] Xia CM , Colomb DG Jr., Akbarali HI, Qiao LY. Prolonged sympathetic innervation of sensory neurons in rat thoracolumbar dorsal root ganglia during chronic colitis. Neurogastroenterol Motil. 2011;23:801-e339. 21605284 10.1111/j.1365-2982.2011.01728.x PMC3282529

[116] Bonaz B , Sinniger V, Pellissier S. Vagal tone: effects on sensitivity, motility, and inflammation. Neurogastroenterol Motil. 2016;28:455–462. 27010234 10.1111/nmo.12817

[117] Ji H et al Central cholinergic activation of a vagus nerve-to-spleen circuit alleviates experimental colitis. Mucosal Immunol. 2014;7:335–347. 23881354 10.1038/mi.2013.52 PMC3859808

[118] Ghia JE , Blennerhassett P, Kumar-Ondiveeran H, Verdu EF, Collins SM. The vagus nerve: a tonic inhibitory influence associated with inflammatory bowel disease in a murine model. Gastroenterology. 2006;131:1122–1130. 17030182 10.1053/j.gastro.2006.08.016

[119] Di Giovangiulio M et al Vagotomy affects the development of oral tolerance and increases susceptibility to develop colitis independently of the alpha-7 nicotinic receptor. Mol Med. 2016;22:464–476. 27341335 10.2119/molmed.2016.00062 PMC5072409

[120] Xie Z et al Enteric neuronal Piezo1 maintains mechanical and immunological homeostasis by sensing force. Cell. 2025;188:2417–2432.e19. 40132579 10.1016/j.cell.2025.02.031 PMC12048284

[121] Zhang W , et al; JRI Live Cell Bank Gut-innervating nociceptors regulate the intestinal microbiota to promote tissue protection. Cell. 2022;185:4170–4189.e20. 36240781 10.1016/j.cell.2022.09.008 PMC9617796

[122] Yang D et al Nociceptor neurons direct goblet cells via a CGRP-RAMP1 axis to drive mucus production and gut barrier protection. Cell. 2022;185:4190–4205.e25. 36243004 10.1016/j.cell.2022.09.024 PMC9617795

[123] Abad C et al Therapeutic effects of vasoactive intestinal peptide in the trinitrobenzene sulfonic acid mice model of Crohn’s disease. Gastroenterology. 2003;124:961–971. 12671893 10.1053/gast.2003.50141

[124] Abad C , Cheung-Lau G, Coute-Monvoisin AC, Waschek JA. Vasoactive intestinal peptide-deficient mice exhibit reduced pathology in trinitrobenzene sulfonic acid-induced colitis. Neuroimmunomodulation. 2015;22:203–212. 25301381 10.1159/000364912 PMC4297532

[125] Voisin T , Bouvier A, Chiu IM. Neuro-immune interactions in allergic diseases: novel targets for therapeutics. Int Immunol. 2017;29:247–261. 28814067 10.1093/intimm/dxx040 PMC5890890

[126] Kokolus KM et al Schweinfurthin natural products induce regression of murine melanoma and pair with anti-PD-1 therapy to facilitate durable tumor immunity. Oncoimmunology. 2019;8:e1539614. 30713799 10.1080/2162402X.2018.1539614 PMC6343772

[127] Duarte Mendes A et al Beta-adrenergic blockade in advanced non-small cell lung cancer patients receiving immunotherapy: a multicentric study. Cureus. 2024;16:e52194. 38348009 10.7759/cureus.52194 PMC10859721

[128] Gandhi S et al Phase I clinical trial of combination propranolol and pembrolizumab in locally advanced and metastatic melanoma: safety, tolerability, and preliminary evidence of antitumor activity. Clin Cancer Res. 2021;27:87–95. 33127652 10.1158/1078-0432.CCR-20-2381 PMC7785669

[129] Li B et al Serotonin transporter inhibits antitumor immunity through regulating the intratumoral serotonin axis. Cell. 2025;188:3823–3842.e21. 40403728 10.1016/j.cell.2025.04.032 PMC12255530

[130] Rosas-Ballina M et al Splenic nerve is required for cholinergic antiinflammatory pathway control of TNF in endotoxemia. Proc Natl Acad Sci U S A. 2008;105:11008–11013. 18669662 10.1073/pnas.0803237105 PMC2504833

[131] Rosas-Ballina M et al Acetylcholine-synthesizing T cells relay neural signals in a vagus nerve circuit. Science. 2011;334:98–101. 21921156 10.1126/science.1209985 PMC4548937

[132] Borovikova LV et al Vagus nerve stimulation attenuates the systemic inflammatory response to endotoxin. Nature. 2000;405:458–462. 10839541 10.1038/35013070

[133] Matteoli G et al A distinct vagal anti-inflammatory pathway modulates intestinal muscularis resident macrophages independent of the spleen. Gut. 2014;63:938–948. 23929694 10.1136/gutjnl-2013-304676

[134] de Jonge WJ et al Stimulation of the vagus nerve attenuates macrophage activation by activating the Jak2-STAT3 signaling pathway. Nat Immunol. 2005;6:844–851. 16025117 10.1038/ni1229

[135] Koopman FA et al Vagus nerve stimulation inhibits cytokine production and attenuates disease severity in rheumatoid arthritis. Proc Natl Acad Sci U S A. 2016;113:8284–8289. 27382171 10.1073/pnas.1605635113 PMC4961187

[136] Peterson D et al Clinical safety and feasibility of a novel implantable neuroimmune modulation device for the treatment of rheumatoid arthritis: initial results from the randomized, double-blind, sham-controlled RESET-RA study. Bioelectron Med. 2024;10:8. 38475923 10.1186/s42234-023-00138-x PMC10935935

[137] Giles JR , Globig AM, Kaech SM, Wherry EJ. CD8(+) T cells in the cancer-immunity cycle. Immunity. 2023;56:2231–2253. 37820583 10.1016/j.immuni.2023.09.005 PMC11237652

[138] Chung HK , McDonald B, Kaech SM. The architectural design of CD8+ T cell responses in acute and chronic infection: parallel structures with divergent fates. J Exp Med. 2021;218:e20201730. 33755719 10.1084/jem.20201730 PMC7992501

[139] Collier JL , Weiss SA, Pauken KE, Sen DR, Sharpe AH. Not-so-opposite ends of the spectrum: CD8(+) T cell dysfunction across chronic infection, cancer and autoimmunity. Nat Immunol. 2021;22:809–819. 34140679 10.1038/s41590-021-00949-7 PMC9197228

[140] Kashem SW et al Nociceptive sensory fibers drive interleukin-23 production from CD301b+ dermal dendritic cells and drive protective cutaneous immunity. Immunity. 2015;43:515–526. 26377898 10.1016/j.immuni.2015.08.016 PMC4607048

[141] Devi S et al Adrenergic regulation of the vasculature impairs leukocyte interstitial migration and suppresses immune responses. Immunity. 2021;54:1219–1230 e1217. 33915109 10.1016/j.immuni.2021.03.025

